# Reese’s Toxicology

**Published:** 1874-05

**Authors:** 


A Manual of Toxicology, including the consideration of the Nature, Properties, Effects, and means of detection of Poisons; more especially in their Medico-Legal Relations. By John J. Reese, M.D., Professor of Medical Jurisprudence and Toxicology in the University of Pennsylvania; Physician to St. Joseph's Hospital, etc., etc. Philadelphia: J. B. Lippincott & Co. 1874. Octavo; Cloth, pp. 507. Price, $5.00 Sold in Atlanta by Phillips & Crew.

Dr. Reese seems likewise to feel that a word of explanation is needed in bringing before the reading public a new work upon Toxicology. "The author is fully aware that the field of investigation on which he has entered has already been very thoroughly explored by others. Names illustrious in the annals of medical and toxicological science are identified with this subject throughout the civilized world. Yet, he ventures to hope that the present treatise will be found a useful text-book, both by the student and the teacher of Toxicology. From an experience of a number of years, he may presume to speak with some authority to the former, as likewise with some degree of confidence to the latter, upon topics with which he is personally familiar. His aim has been to convey his meaning in the simplest style and phraseology, believing this to be the most efficient method of teaching others."

We are pleased with the materials and method of the work, and regard it as well suited to the wants of students of medicine.

